# Temperature- and Diet-Induced Plasticity of Growth and Digestive Enzymes Activity in Spongy Moth Larvae

**DOI:** 10.3390/biom13050821

**Published:** 2023-05-11

**Authors:** Jelica Lazarević, Slobodan Milanović, Darka Šešlija Jovanović, Milena Janković-Tomanić

**Affiliations:** 1 Institute for Biological Research “Siniša Stanković”—National Institute of Republic of Serbia, University of Belgrade, Bulevar Despota Stefana 142, 11060 Belgrade, Serbia; darka.seslija@ibiss.bg.ac.rs (D.Š.J.); miljan@ibiss.bg.ac.rs (M.J.-T.); 2 Faculty of Forestry, University of Belgrade, Kneza Višeslava 1, 11030 Belgrade, Serbia; slobodan.milanovic@sfb.bg.ac.rs; 3 Faculty of Forestry and Wood Technology, Mendel University in Brno, Zemĕdĕlská 3, 613 00 Brno, Czech Republic

**Keywords:** *Lymantria dispar*, gypsy moth, temperature, dietary proteins and carbohydrates, development duration, larval mass, digestive enzymes

## Abstract

Temperature and food quality are the most important environmental factors determining the performance of herbivorous insects. The objective of our study was to evaluate the responses of the spongy moth (formerly known as the gypsy moth) [*Lymantria dispar* L. (Lepidoptera: Erebidae)] to simultaneous variation in these two factors. From hatching to the fourth instar, larvae were exposed to three temperatures (19 °C, 23 °C, and 28 °C) and fed four artificial diets that differed in protein (P) and carbohydrate (C) content. Within each temperature regime, the effects of the nutrient content (P+C) and ratio (P:C) on development duration, larval mass, growth rate, and activities of digestive proteases, carbohydrases, and lipase were examined. It was found that temperature and food quality had a significant effect on the fitness-related traits and digestive physiology of the larvae. The greatest mass and highest growth rate were obtained at 28 °C on a high-protein low-carbohydrate diet. A homeostatic increase in activity was observed for total protease, trypsin, and amylase in response to low substrate levels in the diet. A significant modulation of overall enzyme activities in response to 28 °C was detected only with a low diet quality. A decrease in the nutrient content and P:C ratio only affected the coordination of enzyme activities at 28 °C, as indicated by the significantly altered correlation matrices. Multiple linear regression analysis showed that variation in fitness traits in response to different rearing conditions could be explained by variation in digestion. Our results contribute to the understanding of the role of digestive enzymes in post-ingestive nutrient balancing.

## 1. Introduction

Temperature and food quality are key factors affecting fitness, physiology, behavior, stress tolerance, and, consequently, population dynamics and geographic distribution of herbivorous insects [1,2,3,4]. To survive and reproduce under spatial and temporal variation of these factors, insects have evolved various strategies that assume adaptive plasticity of physiological functions underlying life history traits. Insects respond to variations in the environmental temperature and food quality with reversible plasticity during acclimation and irreversible changes in physiological processes that may affect subsequent developmental stages and generations [5,6,7,8]. A heterogeneous nutritional environment challenges herbivorous insects to adjust their nutrient intake and utilization to needs related to developmental stage, sex, and mating status, as well as the presence of other stressors including suboptimal and supraoptimal temperatures [9,10,11,12,13,14,15].

Laboratory studies have shown that the significant influence of host plants and chemically defined artificial diets have a significant effect on insect development time, larval/pupal mass, and growth rate [16,17,18,19,20,21,22]. These studies indicate that both the content and ratio of nutrients and/or secondary metabolites are important. Of the nutrients, the influences of protein (P) and carbohydrate (C) content and ratios on insect performance have been studied in detail because carbohydrates play an important role as an energy source for metabolism and proteins have multiple functions as enzymes, hormones, stress proteins, building blocks for somatic growth, and substrates for fueling energy metabolism. These nutrients have a major impact on insect life history traits [18,23,24,25,26], consumption and digestive efficiency [18,27,28], metabolite pools [18,29], immune functions [30], and gene expression [31,32].

Achieving maximal performance/fitness depends on an appropriate ratio of ingested nutrients, which usually does not match the ratio in the food [4]. To overcome a nutrient imbalance, insects use various pre- and post-ingestive mechanisms [33]. When given a choice, they self-select between foods of different compositions [34]. Lepidopteran larvae, for example, have an advantage when feeding on multiple hosts [35,36,37], and in experiments with artificial diets, fitness is usually maximized after the ingestion of nutrients that are close to their intake target [23]. However, when there is no choice (e.g., due to low insect mobility or depletion of suitable hosts), insects are forced to use nutritionally poor and/or imbalanced food and must regulate nutrient acquisition at the level of consumption, digestion, absorption, and metabolic conversion [33,38]. For example, insects increase food consumption to compensate for low nutrient content [7,39,40]. This adaptive response comes at a cost when the diet is imbalanced, as energy should be used for physiological mechanisms that mitigate the consequences of excessive intake of carbohydrates when the P:C ratio is low or proteins when the P:C ratio is high. Excess sugars can be converted to stored lipids [32,41], whereas excess ingested proteins can be regulated by reduced secretion of proteolytic enzymes [42], the conversion of amino acids to energy via gluconeogenesis [40], or the excretion of waste products of nitrogen metabolism [43,44].

Exposure of insects to a range of temperatures has significant effects on survival, development time, body mass, longevity, reproduction, and population growth, as well as mobility, consumption, digestion, respiration rate, metabolite pools, lipid composition of membranes, and immune functions [45,46,47,48,49,50,51,52]. In general, development duration shortens with increasing temperature, while body size and growth rate are maximized at moderate temperatures. Adaptive physiological and biochemical responses to suboptimal and supraoptimal temperatures allow the insect to survive stressful conditions [53,54,55]. However, under more intense or prolonged temperature stress, resource depletion and oxidative damage to macromolecules lead to fitness deterioration [3,56,57,58].

A separate assessment of the effects of temperature and diet provided insight into the regulatory mechanisms by which insects cope with environmental heterogeneity. However, because insects live in a multifactorial world in which many interacting factors shape their fitness and physiology [59], the consequences of simultaneous exposure of insects to temperature and nutrient variation must be evaluated. Temperature can affect both the maximum growth rate and the relationship between growth and resource availability [60]. Therefore, the pattern of thermal reaction norms may depend on the quality of food, as the energy requirements of organisms vary with temperature [1]. Such a relationship was confirmed by a significant interaction between temperature and diet in studies of insects reared on plant or artificial diets [61,62,63,64,65,66,67,68].

In the present study, we varied the temperature and the protein and starch content of the diet to investigate how continuous feeding of nutrient-rich vs. poor or protein-biased vs. carbohydrate-biased food shape thermal reaction norms of fitness-related traits and digestive enzyme activities in fourth-instar spongy moth larvae (SML) (*Lymantria dispar* L.). SML was exposed to three temperatures and fed four artificial diets that differed in protein and carbohydrate content or ratio. We recorded the development duration, body mass, growth rate, and activities of midgut enzymes involved in the digestion of proteins (total protease activity, trypsin, elastase, and leucine aminopeptidase), carbohydrases (α-amylase and α-glucosidase), and lipids (lipase).

The spongy moth (SM) is a suitable model organism for studying the interaction effects of temperature and food quality because it is a highly polyphagous cosmopolitan insect and is widely distributed in the Northern Hemisphere [69]. Previously, the interaction effects of temperature and diet quality on SML life history traits, food conversion efficiency, and approximate digestibility have been studied [70,71,72]. However, this is the first study to examine the influence of complex environmental variation on the digestive enzymes of SML. We asked the following research questions: (1) How do different temperatures affect SML growth and digestion? (2) Is the sensitivity of SML to temperature variation affected by the nutrient content and/or nutrient ratio of the diet? (3) How do larvae cope with variations in diet quality at the level of digestion? (4) Are trait correlations sensitive to temperature and/or diet quality? (5) Can variations in digestive enzyme activity in response to temperature and dietary treatments explain the variations in fitness-related traits?

## 2. Materials and Methods

### 2.1. Insect Rearing

Twenty SM egg masses were collected from a poplar forest (*Populus x euramericana* (Dode) Guinier cl. I-214) in Opovo, Serbia (45°03′08″ N and 20°25′49″ E). Average daily maximum temperatures of 18 °C, 23 °C, and 27 °C were recorded at this site in April, May, and June, respectively. Hairs were mechanically removed and eggs were surface-sterilized in 0.1% sodium hypochlorite and laid out to hatch at 23 °C, 12:12 L:D cycle, and 70% RH. After hatching, larvae were released into Petri dishes (9 cm diameter) at a density of 10 larvae per dish in the 1st and 2nd instars, 5 larvae per dish at the 3rd instar, and 1 larva per dish after molting into the 4th instar. Larvae were provided with fresh food cubes daily. Immediately after hatching, larvae were randomly assigned to 12 experimental groups.

### 2.2. Experimental Groups

An experimental design with three temperatures and four artificial diets was used. Larvae were reared at temperatures of 19 °C, 23 °C, and 28 °C. Based on the body mass measured at the beginning of the 4th instar (19 °C: 117.38 ± 3.71 mg; 23 °C: 143.05 ± 4.10 mg; 28 °C: 106.68 ± 4.00 mg), we considered the temperature of 23 °C to be optimal. Within each temperature, larvae were fed 4 diets that differed in protein and carbohydrate content (Table 1). Diets were prepared according to Lindroth et al. [70], who modified a high wheat germ diet [73] to vary the amount of protein (casein). Carbohydrates (potato starch) were added according to Stockhoff [74]. In diets low in protein and carbohydrate content, casein and potato starch were replaced by α-cellulose to keep dry ingredients constant.

Considering that row wheat germ contains 28.56% proteins, 13.54% free sugars, and 7.16% starch (20.70% of carbohydrates) [75,76], the HpHc diet had almost three times higher P+C content than the LpLc diet (45.83 vs. 15.58%), while the P:C ratio was the same (1.02). HpLc and LpHc diets had similar P+C content (30.84 vs. 30.57%), but the P:C ratio was much higher in HpLc (3.00) than in LpHc (0.35). Therefore, by comparisons between HpHc and LpLc groups, we could evaluate the responses of SM to dietary dilution, whereas comparisons between HpLc and LpHc groups showed the responses to the dietary P:C ratio.

### 2.3. Determination of Fitness-Related Traits

Larval molting was monitored daily, and the development duration was measured as the period from hatching to molting into the 4th instar. Larvae were weighed on the 3rd day of the 4th instar and then killed to determine enzyme activities. The growth rate was calculated as the quotient of the larval mass and the time from hatching to the 3rd day of the 4th instar. Fifteen larvae per experimental group were analyzed. For each larva, three fitness-related traits and the activities of seven digestive enzymes were determined.

### 2.4. Enzyme Assays

The activities of total proteases, trypsin, elastase, leucine aminopeptidase, α-amylase, α-glucosidase, and lipase were determined in the crude midgut homogenates. The midguts were dissected on ice and weighed. Crude midgut homogenates were obtained after homogenization in ice-cold 0.9% saline (NaCl) (100 mg tissue/mL) and then centrifuged at 10,000× *g* for 20 min at 4 °C. The supernatants were used for the determination of enzyme activities. The protein content of the supernatants was estimated using the Bradford assay [77], and specific enzyme activities were expressed in enzyme units (U) per mg of proteins. Protein and enzyme assays were performed in duplicate.

Total protease activity was determined according to Kunitz [78] with 1% casein as a substrate. The reaction was carried out in 0.2 M Gly/NaOH (pH 10) buffer at a temperature of 40 °C and stopped after 1 h via the addition of 10% trichloroacetic acid. The amount of aromatic amino acids, tryptophan, and tyrosine released by cleavage of the substrate was spectrometrically measured at 280 nm. A protease unit was defined as an increase in unit optical density at 280 nm per minute.

Trypsin, elastase, and leucine aminopeptidase activities were determined according to the method of Erlanger et al. [79] and Valaitis [80]. N-Benzoyl-DL-arginine-p-nitroanilide (BApNA), N-succinyl-Ala-Ala-Pro-Leu-p-nitroanilide (SA_2_PLpNA), and L-leucine-p-nitroanilide (LpNA) at a concentration of 2 mM were used as substrates for trypsin, elastase, and leucine aminopeptidase, respectively. The reaction mixture contained 50 mM Gly-NaOH (pH 10) buffer for trypsin and elastase and 40 mM veronal/HCl (pH 7.8) buffer for leucine aminopeptidase. The reaction was run at 30 °C for 30 min and stopped with 10% glacial acetic acid. The absorbance of the released p-nitroanaline was measured at 405 nm. One enzyme unit was defined as the amount of enzyme releasing 1 µmol of p-nitroaniline per minute.

α-Amylase was assayed according to a method described by Bernfeld [81] and modified by Doane [82] and Lazarević et al. [83] using 1% soluble starch dissolved in 50 mM Gly-NaOH (pH 9.6) as a substrate. After 30 min at 45 °C, the reaction was stopped with a 3,5-dinitrosalicylic acid reagent. Maltose, the product of starch digestion, was determined by absorbance at 550 nm. One enzyme unit corresponded to 1 µmol of maltose released per minute.

α-Glucosidase was determined by the method of Baker [84] based on the release of p-nitrophenol from the substrate p-nitrophenyl-α-D-glucopyranoside. The substrate and enzyme extract were incubated in 0.2 M sodium phosphate buffer (pH 6) for 10 min at 30 °C, and the reaction was terminated with a mixture of 0.25 M Na_2_CO_3_, 0.25 M NaHCO_3_, and 1% SDS. p-Nitrophenol was determined by absorbance at 405 nm. One enzyme unit was defined as the amount of enzyme releasing 1 µmol of p-nitrophenol per minute.

Lipase activity was measured using p-nitrophenyl caprylate (pNPC) as a substrate according to the method of Arreguin-Espinosa et al. [85], modified from Mrdaković et al. [86]. The enzyme extract and substrate were incubated in 50 mM Tris-HCl buffer (pH 8.2) for 3 min at 37 °C, and the change in absorbance was measured continuously at 410 nm. One unit of enzyme activity was defined as the amount of enzyme releasing 1 µmol of p-nitrophenol per minute.

### 2.5. Statistical Analyses

To reveal the main and interaction effects of temperature and diet quality on fitness-related traits and digestive enzyme activities, Statistica 13 (TIBCO Software Inc., Palo Alto, CA, USA) was used to perform multivariate and univariate 2-way ANOVAs on square root-transformed values of the development duration, log-transformed values of larval mass on the 3rd day of larval instar, the growth rate, protease, trypsin, elastase, leucine aminopeptidase, and amylase activities, and untransformed values of α-glucosidase and lipase activities. The Tukey HSD test was applied to compare the average effects of the two factors, and a posteriori comparisons (contrasts) of least square trait means (LSM) were applied for the evaluation of significant differences between temperatures within each diet and between diets within each temperature group. Three-way ANOVAs were also performed with temperature, protein, and carbohydrate content as factors.

Pearson correlation coefficients between fitness-related traits and between enzyme activities within different treatments were determined. To determine how different temperatures and dietary nutrient content and ratios affect the coordination of enzyme activities, correlation matrices were compared with the Mantel test performed using PAST version 4.03 software. For comparisons between specific correlation coefficients, the z-test was used. Since 21 comparisons were made, the critical z value for *p* = 0.0024 (two-sided) was 3.03.

Multivariate analysis of variance (MANOVA) was followed by canonical discriminant analysis (CDA), which tested the overall differences in fitness-related traits and enzyme activities between treatments. The growth rate was not included in the MANOVA and CDA. The F-test was applied to determine the significance of squared Mahalanobis distances between group centroids.

To relate the larval development duration, body mass, and growth rate to variation in digestive enzyme activities in response to temperature and diet quality, we performed backward stepwise multiple regression (*p* = 0.1 for trait selection and removal) on the trait means of each of the 12 experimental groups.

## 3. Results

### 3.1. Influence of Rearing Temperature and Diet Quality on Larval Fitness-Related Traits

Significant effects of temperature and diet quality (Temperature: Pillai’s trace = 0.896, F_4,336_ = 68.21, *p* < 0.001; Diet: Pillai’s trace = 0.325, F_6,336_ = 10.88, *p* < 0.001) and their independent influence on development duration and body mass (T × D: Pillai’s trace = 0.025, F_12,336_ = 0.35, *p* = 0.979) were demonstrated by MANOVA. Differences in dietary proteins were the main factor for the significant effect of diet quality, while the contribution of carbohydrate content and the P × C interaction were only marginally significant (Table S1). Univariate tests confirmed that both fitness-related traits contributed to the differences between experimental groups.

**Figure 1:**
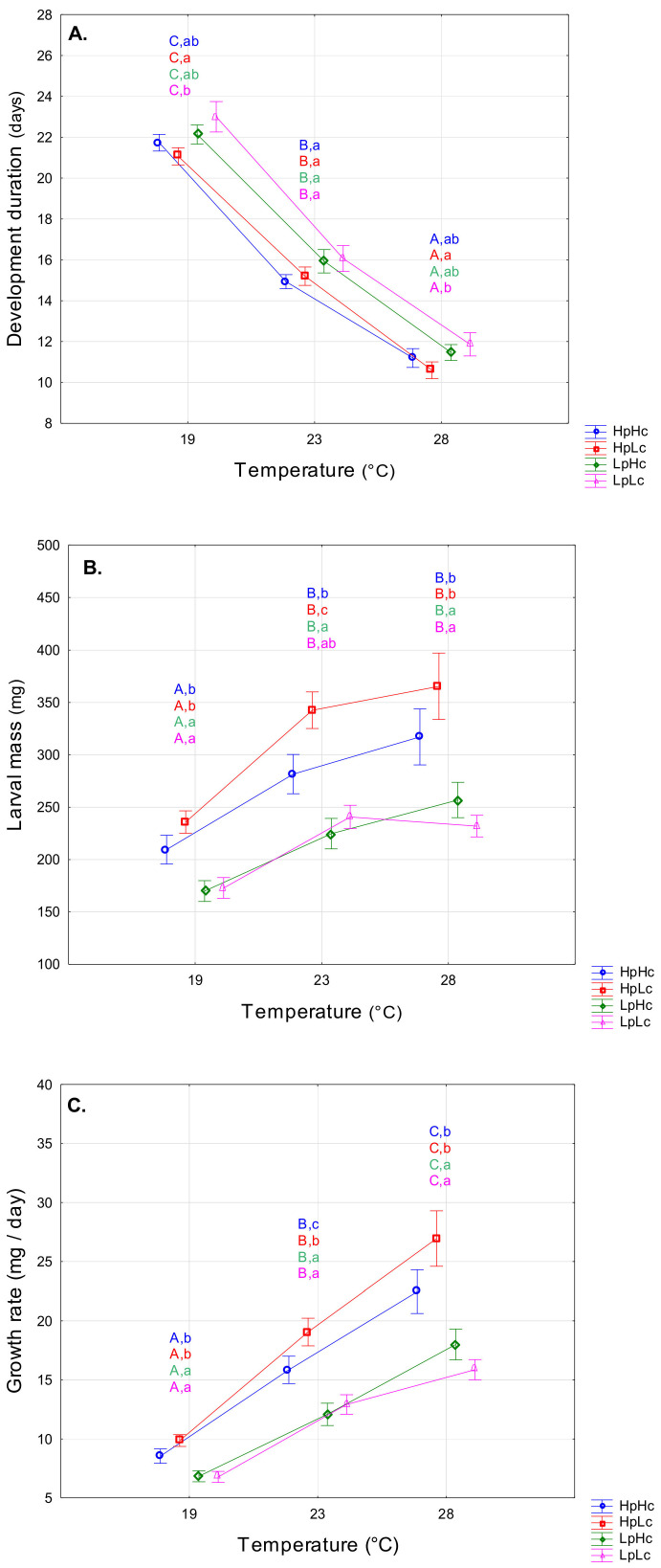
Means and standard errors (SE) for development duration up to molting into the fourth instar (**A**), larval mass on the third day of the fourth instar (**B**) and growth rate (**C**) in spongy moth larvae reared at 19 °C, 23 °C, and 28 °C and fed on four artificial diets that contained different amounts of proteins (Hp—high content and Lp—low content) and carbohydrates (Hc—high content and Lc—low content). Different capital letters mark significant trait differences among temperatures within each diet and small letters mark significant differences among diets within each temperature (LSM contrasts, *p* < 0.05).

Development duration was significantly affected by the temperature (F_2,168_ = 463.60, *p* < 0.001) and the diet type (F_3,168_ = 4.07, *p* = 0.008). Dietary effects resulted from differences in protein content (Table S1). The decrease in development duration with temperature elevation was similar across all four diets (Figure 1A), resulting in a non-significant interaction term in the 2-way ANOVA (F_6,168_ = 0.25, *p* = 0.961). On average, larvae reared on diets with low carbohydrate content took longer to develop when protein content was also low (LpLc > HpLc) (*p* = 0.007). Such a relationship was observed at temperatures of 19 °C and 28 °C, while no differences in development duration between diets were recorded at 23 °C (Figure 1A).

Temperature (T) and diet quality (D) exhibited a significant and independent effect on larval mass (T: F_2,168_ = 43.45, *p* < 0.001; D: F_3,168_ = 24.37, *p* < 0.001; T × D: F_6,168_ = 0.43, *p* = 0.8602). On average, larvae reared at 19 °C were smaller than larvae reared at 23 °C and 28 °C (*p* < 0.001). The best performance was recorded on a protein-biased diet. Namely, on the third day, HpLc larvae were heavier than HpHc (*p* = 0.010), LpHc (*p* < 0.001), and LpLc (*p* < 0.001). It is obvious that a high-protein diet is beneficial for the growth of SML and that the ratio of proteins to carbohydrates becomes important at an optimal temperature of 23 °C (Figure 1B). Three-way ANOVA also confirmed that high protein content and low carbohydrate content favored high growth (significant P and C terms in Table S1).

The growth rate (GR) increased gradually with temperature in all diets studied, and similar to the results on body mass, the pattern of increase was independent of dietary influences (T: F_2,168_ = 189.79, *p* < 0.001; D: F_3,168_ = 26.08, *p* < 0.001; T × D: F_6,168_ = 0.34, *p* = 0.914) (Figure 1C). The increase in GR was most pronounced at 28 °C. On average, the fastest growth was observed with the HpLc diet and the slowest growth with the two low-protein diets. In addition to the significant temperature effect, a three-way ANOVA revealed significant protein, marginally significant carbohydrate, and significant P × C terms (P: F_1,168_ = 68.50, *p* < 0.001; C: F_1,168_ = 3.88, *p* = 0.051; P × C: F_1,168_ = 5.87, *p* = 0.016). Other terms were not significant (T × P: F_2,168_ = 0.12, *p* = 0.887; T × C: F_2,168_ = 0.54, *p* = 0.586; T × P × C: F_2,168_ = 0.37, *p* = 0.691).

Temperature and dietary treatments did not affect the correlations between the development duration and larval body mass. Across all experimental groups, body mass was negatively correlated with the development duration (r = −0.525, *p* < 0.05). However, within treatments, none of the correlations were significant (19 °C: r-_HpHc_ = −0.317, r-_HpLc_ = −0.157, r-_LpHc_ = −0.390, r-_LpLc_ = −0.436; 23 °C: r-_HpHc_ = −0.239, r-_HpLc_ = −0.264, r-_LpHc_ = −0.183, r-_LpLc_ = −0.399; 28 °C: r-_HpHc_ = 0.219, r-_HpLc_ = 0.314, r-_LpHc_ = −0.204, r-_LpLc_ = 0.076).

Canonical discriminant analysis of development duration and body mass showed that two canonical functions significantly accounted for the differentiation among 12 experimental groups. They explained 93.7% (R^2^ = 0.854) and 6.3% (R^2^ = 0.283) of the variance, respectively. Both the two functions in combination (Chi-square = 387.94, df = 22, *p* < 0.001) and the second dimension alone (Chi-square = 57.21, df = 10, *p* < 0.001) can explain group differences. Overall, there was a significant group differentiation among temperature treatments within each diet and between protein- and carbohydrate-biased groups at all temperatures examined (Table S2A,B). However, larval fitness only responded to diet dilution at 28 °C (Table S2B).

**Figure 2:**
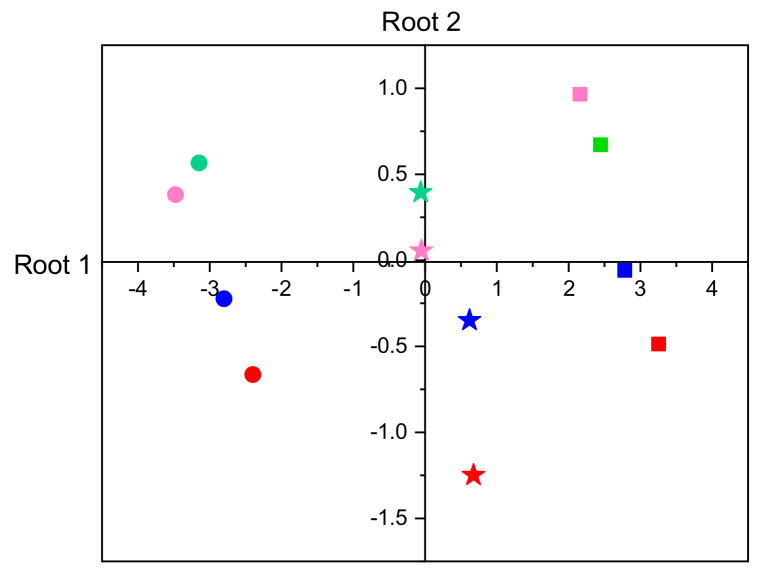
Discrimination of experimental groups by canonical discriminant analysis based on larval development duration and larval mass. Canonical means (centroids) are presented for diets HpHc, HpLc, LpHc, and LpLc as blue, red, green, and pink, respectively; and for temperatures of 19 °C, 23 °C, and 28 °C as circles, asterisks, and squares, respectively.

Within-group canonical structure coefficients revealed that development duration and larval mass had an opposite relationship with root 1 and a similar relationship with root 2 (Table 2). It was also clear that development duration contributed strongly to group differentiation on root 1, while larval mass contributed the most to group separation on root 2. Discriminant function plots (Figure 2) showed that the first function differentiated temperature groups, while the second function differentiated diet groups (HpHc vs. LpLc and HpLc vs. LpHc).

### 3.2. Influence of Rearing Temperature and Diet Quality on Activities of Digestive Enzymes

MANOVA revealed that temperature and diet quality had a significant and independent effect on digestive enzyme activities (T: Pillai’s trace = 0.828, F_4,336_ = 16.44, *p* < 0.001; D: Pillai’s trace = 0.762, F_6,336_ = 7.98, *p* < 0.001; T × D: Pillai’s trace = 0.316, F_12,336_ = 0.35, *p* = 0.979). A three-way ANOVA showed a significant average response across digestive enzyme activities to protein and carbohydrate content and a dependence of thermal reaction norms on protein content (significant T × P term in Table S2) and carbohydrate content (marginally significant T × C term in Table S2). Univariate tests (see below) again showed that most enzyme activities contributed to the differences between the temperature and diet groups.

#### 3.2.1. Proteolytic Enzymes

**Figure 3:**
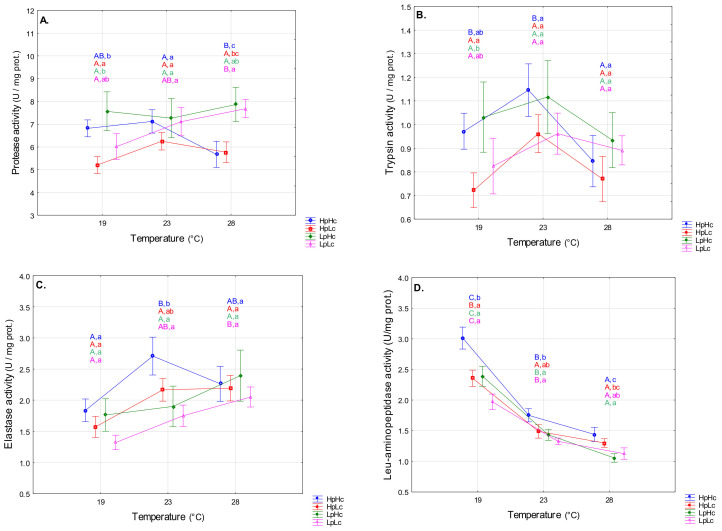
Specific activities (means ± SE) of total protease (**A**), trypsin (**B**), elastase (**C**), and leucine aminopeptidase (**D**) in midgut of the fourth instar spongy moth larvae reared at 19 °C, 23 °C, and 28 °C and fed four artificial diets that contained different amounts of proteins (Hp—high content and Lp—low content) and carbohydrates (Hc—high content and Lc—low content). Different capital letters point to significant activity differences among temperatures within each diet and small letters mark significant differences among diets within each temperature (LSM contrasts, *p* < 0.05).

The activities of trypsin (F_2,168_ = 3.57, *p* = 0.030), elastase (F_2,168_ = 6.71, *p* = 0.002), and leucine aminopeptidase (F_2,168_ = 107.08, *p* < 0.001) were sensitive to temperature variation in contrast to total protease activity (F_2,168_ = 0.87, *p* = 0.420). On average, elastase activity increased, and leucine aminopeptidase decreased with temperature, while trypsin activity was highest at 23 °C (Figure 3B–D). The influence of diet quality on digestive enzyme activity was significant (total protease: F_3,168_ = 2.80, *p* = 0.041; leucine aminopeptidase: F_3,168_ = 11.59, *p* < 0.001) or marginally significant (trypsin: F_3,168_ = 2.38, *p* = 0.072; elastase: F_3,168_ = 2.15, *p* = 0.096). The effect of diet composition was not dependent on rearing temperature (total protease: F_6,168_ = 1.74, *p* = 0.114; trypsin: F_6,168_ = 0.33, *p* = 0.918; elastase: F_6,168_ = 0.68, *p* = 0.664; leucine aminopeptidase: F_6,168_ = 0.92, *p* = 0.483). A three-way ANOVA revealed higher activities of total protease and elastase in low- than high-protein diets (significant P term in Table S3), while leucine aminopeptidase activity was lower when both protein content and carbohydrate content of the diet was low (significant P and C terms in Table S3). On average, total protease and trypsin activities were lower in a protein-biased diet (HpLc) than in a carbohydrate-biased diet (LpHc), and leucine aminopeptidase activity was higher on high-protein diets than on low-protein diets (Figure 3A,B,D). Planned comparisons revealed a significant decrease in total protease, trypsin, and elastase activities in response to a temperature deviation from 23 °C only on the nutrient-rich diet HpHc (Figure 3A–C), whereas a decrease in leucine aminopeptidase was observed in all diets studied (Figure 3D). Lower activity of total protease (28 °C) and higher activity of elastase (23 °C) and leucine aminopeptidase (19 °C, 23 °C, and 28 °C) were observed in the HpHc group than in the LpLc group (Figure 3A,C,D). At a temperature of 19 °C, total protease and trypsin activities were lower in the HpLc group than in the LpHc group (Figure 3A,B).

#### 3.2.2. Carbohydrases

**Figure 4:**
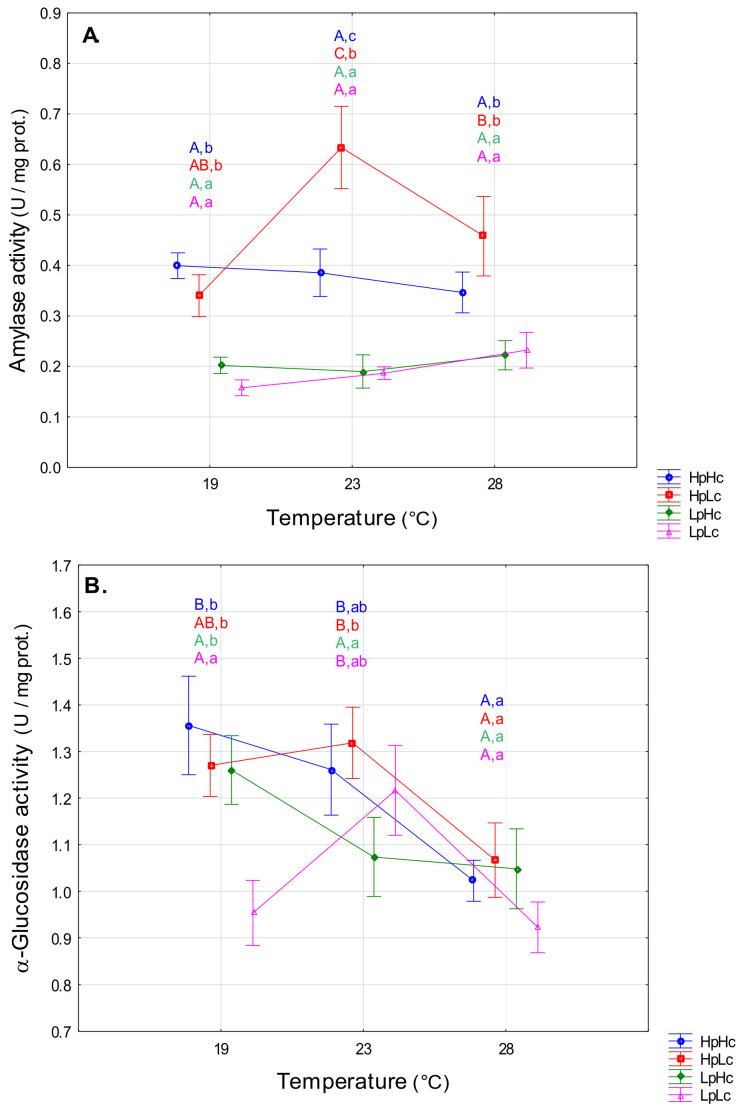
Specific activities (means ± SE) of amylase (**A**) and α-glucosidase (**B**) in the midgut of the fourth instar spongy moth larvae reared at 19 °C, 23 °C, and 28 °C and fed four artificial diets that contained different amounts of proteins (Hp—high content and Lp—low content) and carbohydrates (Hc—high content and Lc—low content). Different capital letters mark significant activity differences among temperatures within each diet and small letters mark significant differences among diets within each temperature (LSM contrasts, *p* < 0.05).

The average effect of temperature on amylase activity was not significant (F_2,168_ = 0.66, *p* = 0.518). However, the variation in thermal reaction norms between diets can be seen in Figure 4A (F_6,168_ = 2.26, *p* = 0.040). Planned comparisons showed that the protein-biased diet induced the highest activity at 23 °C, whereas larvae fed other diets did not significantly alter amylase activity as a function of temperature (Figure 4A). The influence of temperature on α-glucosidase activity was highly significant (F_2,168_ = 8.27, *p* < 0.001). On average, activity was reduced at the highest temperature. Planned comparisons revealed that a temperature decrease from 23 °C to 19 °C significantly decreased glucosidase activity on the LpLc diet (*p* = 0.021), while a marginally significant activity increase was recorded on the LpHc diet (*p* = 0.099). An increase in temperature from 23 °C to 28 °C decreased glucosidase activity in all diet groups except LpHc (Figure 4B).

Diet quality significantly affected both amylase (F_3,168_ = 25.74, *p* < 0.001) and α-glucosidase activity (F_3,168_ = 3.67, *p* = 0.013). On average, amylase activity was significantly higher on HpHc than on LpLc and on HpLc than on LpHc. On the other hand, similar α-glucosidase activity was recorded in these groups. Amylase activity was higher on high-protein diets than on low-protein diets at all temperatures studied (Figure 4A), and α-glucosidase activity was higher at 19 °C (Figure 4B). The α-glucosidase activity was higher at 19 °C in the nutrient-rich diet than in the nutrient-poor diet (HpHc vs. LpLc) and at 23 °C on the protein-biased diet than on the carbohydrate-biased diet (HpLc vs. LpHc). A three-way ANOVA revealed a significant effect of protein content on carbohydrases (Table S3). The pattern of thermal reaction norms of these enzymes also depended on the carbohydrate content (significant T × C term in Table S3). In diets with a low carbohydrate content, the maximum average activity was measured at 23 °C, whereas at a high carbohydrate content, the activity of carbohydrases decreased from 19 °C to 28 °C.

#### 3.2.3. Lipase

**Figure 5:**
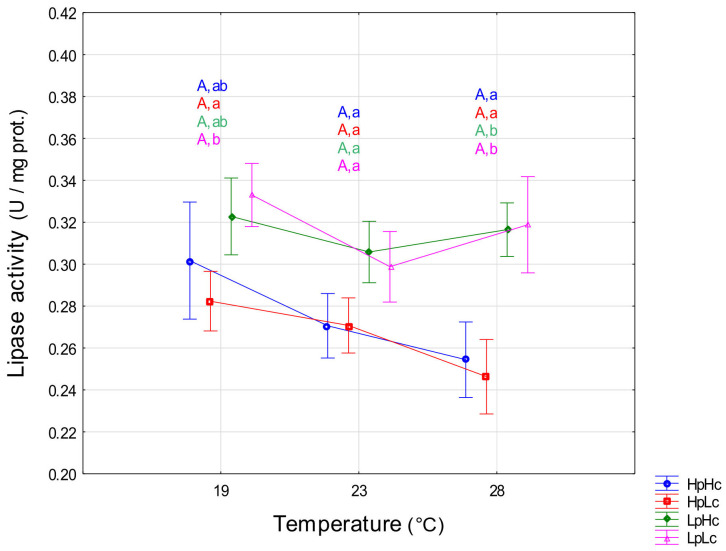
Specific lipase activity (means ± SE) in midgut of fourth instar spongy moth larvae reared at 19 °C, 23 °C, and 28 °C and fed four artificial diets that contain different amounts of proteins (Hp—high content and Lp—low content) and carbohydrates (Hc—high content and Lc—low content). Different capital letters mark significant activity differences among temperatures within each diet and small letters mark significant differences among diets within each temperature (LSM contrasts, *p* < 0.05).

There was a marginally significant effect of temperature with a trend toward a decrease in mean lipase activity with increasing temperature (F_2,168_ = 2.60, *p* = 0.077). The effect of diet quality on lipase activity was significant (F_3,168_ = 6.53, *p* < 0.001). On average, activity was lower on the HpHc diet than on the LpLc diet (*p* = 0.023) and on the HpLc diet than on the LpHc diet (*p* = 0.005). Planned comparisons showed that activity at 28 °C was higher on low-protein diets than on high-protein diets, whereas at 23 °C, diet quality did not significantly affect lipase activity (Figure 5). Protein content was the most important factor in modulating lipase activity by diet (Table S3).

#### 3.2.4. Correlations among Enzyme Activities

**Figure 6:**
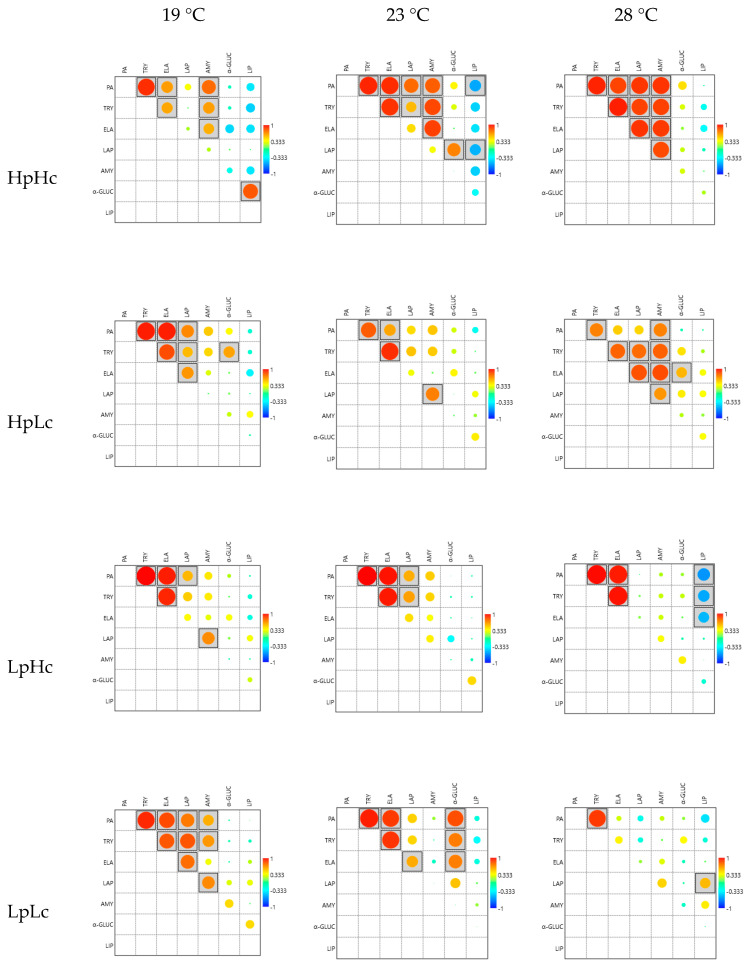
Color maps of Pearson’s correlations among digestive enzyme activities in the midgut of fourth instar spongy moth larvae reared at 19 °C, 23 °C, and 28 °C and fed four artificial diets that contained different amounts of proteins (Hp—high content and Lp—low content) and carbohydrates (Hc—high content and Lc—low content). Correlations vary within the range of +1 (red) to −1 (blue). Significant correlations (*p* < 0.05) are given in boxes. PA—total protease activity, TRY—trypsin, LAP—leucine aminopeptidase, AMY—leucine aminopeptidase, α-GLUC—α-glucosidase, LIP—lipase.

Figure 6 shows the color maps of the correlations between the activities of the digestive enzymes. It can be seen that the increase in temperature from 19 °C to 28 °C increases the number of significant correlations for all diets except the nutrient-poor diet. Significant correlations between proteolytic enzymes and between proteolytic enzymes and carbohydrases were always positive. Significant negative correlations were found between proteolytic enzymes and lipase in larvae reared at 23 °C on a nutrient-rich diet and in larvae reared at 28 °C on a carbohydrate-biased diet. The correlation structure was largely similar between experimental groups. An increase in temperature from 23 °C to 28 °C significantly affected the correlation structure only on the nutrient-poor diet (Table S4A). Furthermore, diet dilution and the nutrient ratio significantly affected the correlation structure but only at 28 °C (Table S4B).

Only four specific correlations differed significantly between experimental groups. In larvae fed the HpHc diet, the increase in temperature from 19 °C to 23 °C changed the correlation between α-glucosidase (α-GLUC) and lipase (LIP) activity from +0.768 to −0.315 (Table S4A). Reducing the nutrient content changed three correlations from strongly significant positive to non-significant negative values. The correlation between elastase (ELA) and amylase (AMY) activity in larvae reared at 23 °C decreased from +0.829 on the HpHc diet to −0.169 on the LpLc diet. Correlations of leucine aminopeptidase (LAP) with total protease (PA) and trypsin (TRY) activity in larvae reared at 28 °C also decreased with diet dilution from +0.850 to −0.274 and +0.823 to −0.242, respectively (Table S4B).

#### 3.2.5. Discriminant Analysis of Enzyme Activities

Discriminant analysis of enzyme activities revealed that seven canonical functions contributed to the variation in digestive enzyme activities among the 12 rearing conditions (Chi-square = 450.62, df = 77, *p* < 0.001). Significant group discrimination remained after the removal of the first (Chi-square = 239.98, df = 60, *p* < 0.001) and second dimensions (Chi-square = 68.83, df = 45, *p* = 0.013), but not after the removal of the third dimension (Chi-square = 40.65, df = 32, *p* = 0.140). The first function explained 53.1% (R^2^ = 0.711), the second function explained 37.5% (R^2^ = 0.636), and the third function explained 3.9% (R^2^ = 0.153) of the data variation. An increase in temperature from 19 °C to 23 °C significantly changed the overall enzyme activities in all diets, whereas a temperature of 28 °C caused significant changes only in the low-protein diets (Table S5A). Regardless of the ambient temperature, both diet dilution and a low P:C ratio significantly affected overall enzyme activities (Table S5B).

**Figure 7:**
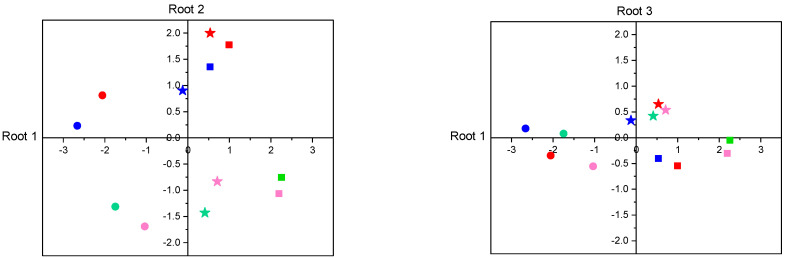
Discrimination of experimental groups by canonical discriminant analysis based on digestive enzyme activities. Means of discriminant scores are presented for diets HpHc, HpLc, LpHc, and LpLc as blue, red, green, and pink, respectively; and for temperatures of 19 °C, 23 °C, and 28 °C as circles, asterisks, and squares, respectively.

Canonical correlations between enzyme activities and discriminant functions (within groups’ loadings) revealed that PA, TRY, and ELA were positively correlated with root 1, whereas LAP, AMY, α-GLUC, and LIP were negatively correlated (Table 3). The strongest correlation was found for LAP (−0.777). Correlations with root 2 showed a positive relationship with ELA, AMY, and α-GLUC and a negative relationship with PA, TRY, LAP, and LIP. AMY had the highest loading on root 2 (+0.517). All enzyme activities had a similar and positive relationship with root 3, which was strongest for TRY (+0.567) and α-GLUC (+0.694). Root 1 discriminated different temperature groups (Figure 7), whereas HpHc vs. LpLc and HpLc vs. LpHc groups differentiated based on root 2 (Figure 7). Root 3 distinguished the HpHc vs. LpLc and HpLc vs. LpHc groups at 19 °C, and 23 °C from 28 °C groups on all diets (Figure 7).

### 3.3. Relationship between Fitness-Related Traits and Digestive Enzyme Activities

Multiple regression analysis showed that a significant proportion of the variation in the SML development duration, body mass, and growth rate could be explained by the variation in digestive enzyme activities (Table 4). Larval development duration was negatively related to total protease activity and positively related to trypsin, leucine aminopeptidase, α-glucosidase, and lipase activity. Protease and lipase activity had the greatest effect on development. Larval body mass on the third day of the fourth instar was strongly positively related to amylase activity and negatively related to leucine aminopeptidase and lipase. All three enzymes had a strong effect on body mass. The growth rate was positively related to total protease and amylase activity and negatively related to trypsin, α-glucosidase, and lipase activity.

## 4. Discussion

As a polyphagous pest, SM encounters high variation in host plant quality. In addition to variations in nutrient and secondary metabolite content among plant species [87,88,89], large within-species differences have also been found, depending on the plant genotype, degree of defoliation, leaf age, or position in the canopy [90,91,92,93,94,95]. SM also faces diurnal and seasonal temperature variations specific to geographic location (latitude, longitude, and altitude). Forest area, annual average temperature, and altitude are among the most important factors determining the occurrence of SM outbreaks [96]. Temperature can affect the performance of SM both directly and indirectly through changes in leaf composition [97,98,99]. Global warming affects synchrony/asynchrony between SML hatching and host plant budding, leading to different scenarios of interaction between temperature and leaf quality that influence larval development, susceptibility to natural enemies, population dynamics, and host plant defoliation [100,101,102,103]. As confirmed by data on SM [99,104,105] and the closely related nun moth [106], another consequence of global warming is the spread of the pest to northern geographic regions where new dominant hosts with different leaf chemistry are encountered. Under these circumstances, the energy balance of food utilization determines survival, host acceptance for feeding, successful development, body size, female fecundity, flight potential of males, and thus invasion dynamics [107,108,109]. Therefore, it is important to study the interdependence of the influences of temperature and food quality. Here, we presented results on the growth and digestive performance of fourth instar larvae exposed to three temperatures and four diets and confirmed that temperature and food quality significantly influenced overall fitness traits and digestive enzyme activities.

### 4.1. Temperature- and Diet-Induced Plasticity of Fitness-Related Traits

The influence of temperature on fitness traits was more evident than the influence of diet. A temperature of 23 °C, which we considered optimal for the development of the early instars of SM, corresponded to the average daily maximum at the Opovo site in May when the fourth larval stage began. At the beginning of the fourth larval stage, larvae were heavier at 23 °C than at 19 and 28 °C. However, the mass of larvae on the third day of instar did not differ between optimal and supraoptimal temperature. It is possible that the later larval stages may cope more successfully with higher temperatures. Results on the heat sensitivity of SM growth may vary from study to study depending on experimental conditions, the developmental stage, and population origin [8,99,110,111,112]. Similar to our results, short-term (3 days) exposure of fifth instar SML to 28 °C resulted in a higher growth rate compared to larvae at 23 °C [8]. High pupal mass was achieved in a range of constant (22–28 °C) or fluctuating temperatures, although the development time was significantly reduced [70,72,113]. These and many other studies [51,52,114,115,116] indicated that shortened development time is a general response of larvae to an increase in temperature. The lack of correlation between development duration and larval mass in our study suggests that the maximization of growth could be achieved regardless of development duration, which is characteristic of outbreaking lepidopterans [117].

Significant separation of temperature groups in fitness traits was based on the first canonical function, to which the development duration made a significant contribution. Larval mass, on the other hand, was highly loaded on the second canonical function, which separated dietary groups with different nutrient contents or ratios. Previous studies have shown that SML had higher mass when fed diets with a high protein content or high protein–sucrose ratio [20,39,70,72], which is consistent with our results and those of other authors on lepidopteran species [40,118]. In natural plant diets, which also contain various secondary metabolites, the nitrogen content of leaves is positively related to the growth rate of SML, while the influence of starch is either positive, negative, or not significant [91,119,120]. In our experiment with artificial diets, we compared SML fitness traits within a wide range of protein (23.14 and 7.88%) and starch (18.96 and 3.97%) contents. Although these values were very high, they were within a range found in the leaves of different host plant species [88,89,94,119,121]. The high protein content of 23% is close to the protein intake target determined for SML [122] and could be considered optimal. We found a non-significant effect of starch on the development duration and a negative effect of starch on larval mass on the third day of the fourth instar. Larvae were larger on high-protein diets when the starch content in the diet was low. Barbehenn et al. [123] studied the efficiency of nutrient assimilation in SML reared on four host plants with different nutrient contents and ratios and found that the efficiency of carbohydrate assimilation increased when the proportion of sugars in total leaf carbohydrates was higher. Our diets had the same sugar content derived only from wheat germ, but diets with high starch content had lower sugar proportion and therefore could provide less energy for larval growth.

As shown by the non-significant interaction terms (T × D, T × P, T × C) in the ANOVAs, thermal reaction norms of the fitness traits were similar among the diets studied and were not dependent on protein or carbohydrate content. In our previous study on a large sample of larvae, we found a marginally significant T × P interaction for larval mass at the beginning of the fourth instar and showed that larvae were more sensitive to low protein content in the diet at 28 °C than at 23 °C [71]. Similarly, here we found large Mahalanobis distances for overall fitness traits (development duration and body mass) between groups fed nutrient-rich and nutrient-poor diets at 28 °C only. Lindroth et al. [70] and Sostak [72] also failed to find a significant T × P interaction for SM larval duration and pupal mass. The results on the SM are in contrast with those obtained in other species in studies examining more different temperatures and/or diets. For example, in *Spodoptera exigua* larvae, a significant T × D interaction was found for the larval duration and growth rate [62]. In that study, six diets with different P:C ratios were examined, and it was found that the increase in growth rate with temperature from 18 to 36 °C was much weaker for two extreme diets with the lowest and highest P:C ratios. In addition, the two extreme diets only significantly affected development duration at 18 °C. Kutz et al. [65] studied up to 36 diets with different contents and ratios of proteins and carbohydrates and found that the response of *Drosophila melanogaster* life history traits to a temperature change from 25 °C to 28 °C was dependent on the protein content of the diet. It may not be possible to capture these effects in the range of dietary proteins commonly found in the natural environment of an insect species. The optimal P:C ratio is species-specific. In *Spodoptera litura*, a carbohydrate-biased diet allowed heavier pupae at 20 °C and 25 °C, while a balanced diet was beneficial at 30 °C [124]. In contrast, faster mass gain was observed in larvae of *S. exigua* at 30 °C than at 25 °C when the diet had a higher P:C ratio [64]. It is suggested that the nitrogen turnover and respiration rate increase at higher temperatures, so the limited dietary proteins cannot meet nutrient requirements, which in turn leads to a significant T × P interaction. SML fed diets with the same high and low protein content as in our study did not differ in respiration rate at 25 °C [74], while increasing the fluctuating temperature from 19:16 °C to 25:22 °C increased the growth rate, assimilation, and growth efficiencies in the fourth instar SML more in diets with high than low protein content.

### 4.2. Temperature- and Diet-Induced Plasticity of Digestive Enzyme Activities

Assimilation efficiency depends on nutrient absorption, the rate of food passage through the gut, and the activity of digestive enzymes. Here we studied the influence of temperature and diet on the activities and correlations of proteases, carbohydrases, and lipase. The digestion of proteins and starch begins in the midgut lumen by the action of endopeptidases (trypsin and elastase), which break the internal bonds of proteins, and α-amylase, which hydrolyzes the internal bonds in long α-1,4-glucan chains of starch [125]. In the spongy moth, serine proteinases are involved in the initial phases of protein digestion, and elastase is the major proteinase [80]. Aminopeptidases and α-glucosidases cleave terminal bonds in the short peptide (oligopeptides) and carbohydrate (oligosaccharides and disaccharides) chains. Lipases hydrolyze triglycerides by releasing fatty acids from the α-position [125]. The resulting 2-monoacylglycerols and fatty acids can be absorbed by the gut epithelium together with glucose released by amylase and glucosidase.

The average response of SML to long-term exposure to temperatures of 19–28 °C from hatching to the fourth instar was reduced lipase activity with increasing temperature and a lack of total protease and amylase plasticity. Zeng et al. [126] recorded a similar SML response of digestive enzymes after short-term (3 h) exposure to temperatures of 20–30 °C and found that protease and amylase activities were reduced only at extreme temperatures of 15 °C and 35 °C. In addition, we documented bell-shaped average thermal reaction norms for endopeptidases, as well as a decrease in expopeptidase (LAP) from 19 °C to 28 °C and exocarbohydrase (α-GLUC) activities from 23 °C to 28 °C. Thermal responses of digestive enzymes are species-specific and depend on exposure time. For example, temperature effects on trypsin (lower at 22 °C than at 32 °C) and lipase (higher at 22 °C than at 32 °C) secretion were more pronounced in adult *Gryllus bimaculatus* after long-term exposure starting at the egg or penultimate instar stage than after short-term 2-day exposure of adults [127,128]. In contrast to our results, in *Helicoverpa armigera*, the increase in larval growth from 15 °C to 35 °C was followed by increased activity of proteases and amylase [129], suggesting that heat tolerance differs among species. The decreased activity observed in our study could be due to altered gene expression or enzyme conformation, which depends on the structural stability of enzyme molecules. To protect against oxidative stress caused by elevated temperatures and to preserve enzyme conformation, SM increases the level of heat shock proteins and the activities of antioxidative enzymes and alkaline phosphatase [8,130]. It is also possible that long-term acclimation to 28 °C during the first three instars reorganizes SML metabolism such that resources are allocated to protective mechanisms rather than to the synthesis of digestive enzymes. Since in our experiment a higher growth rate of larvae at 28 °C than at 23 °C was followed by reduced activity of several digestive enzymes, it seems that other adaptive responses such as improved nutrient absorption or more efficient conversion of absorbed nutrients could support the increase in larval growth. Indeed, Lindroth et al. [70] found that the nitrogen accumulation rate and utilization efficiency increased with temperature in SML.

The activities of digestive enzymes are related to the adaptation of an insect species to specific food composition, whereas the adaptive dietary modulation of digestive enzymes within insect species reflects the ability to utilize different plants and expand the host range. The gut epithelium, which produces digestive enzymes and transporters for the absorption of nutrient digestion products, is highly regulated and responsive to both dietary components and neuroendocrine signals involved in matching the nutrient intake to an organism’s nutrient requirements [131,132,133]. A significant or marginally significant effect of diet quality was found for all enzymes studied. Diet dilution decreased leucine aminopeptidase and amylase at all temperatures, elastase at 23 °C, and α-glucosidase at 19 °C. Similarly, the black soldier fly decreased amylase and lipase activity on low-starch and low-fat diets [134]. The adjustment of enzyme activities to substrate levels in balanced diets represents an adaptive response that avoids the costly production of enzyme proteins. On the other hand, the increase in total protease and lipase activity at 28 °C may be directed toward providing amino acids and energy for growth and meeting increased energy demands at higher temperatures. In the fourth instar SML, a reduction in dietary protein content at 25 °C resulted in a 13.7% increase in body carbohydrates and had no effect on the respiration rate or lipid accumulation [74]. Studies in other insect species confirmed that the importance of diet composition increases with temperature, as the respiration rate increases when either protein or carbohydrate becomes the limiting nutrient [64,134]. On high- and low-P:C-ratio diets, we observed a homeostatic modulation of digestive enzymes that contributed to nutrient balancing. Larvae responded to a lower P:C ratio with a decrease in amylase at all temperatures and α-glucosidase activity at 23 °C, whereas protease and trypsin activities were increased at 19 °C. Moreover, the activity of lipase was increased only at the highest temperature. Therefore, we found significant changes in enzyme activities as a function of both the nutrient content and ratio, whereas research on other insect species showed that either the nutrient content [31] or ratio was more important [42].

Our previous report on SML, which fed on two oak species, showed that amylase was induced on leaves with lower starch content [6]. Moreover, switching from oak to beech leaves containing less nitrogen induced trypsin activity and had no effect on approximate digestibility [135,136]. The importance of protein content and a high P:C ratio for the growth and development of SML is well documented. To compensate for low dietary nitrogen, larvae consume more food and manage to maintain constant growth until nitrogen levels drop below 3%, when gross growth efficiency declines mainly due to lower approximate digestibility [39,122]. This is consistent with the lower activities of various digestive enzymes observed in our study on a nutrient-deficient diet. On isocaloric diets, SML responds to a decrease in the P:C ratio from 2 to 0.5 with the prolongation of larval instar, lower consumption, and higher approximate digestibility, while larval fresh mass did not change [20]. Despite the adaptive modulation in enzyme activities that we observed when comparing larvae fed diets with P:C ratios of 3 and 0.35, larval mass was significantly reduced on the diet with a low P:C ratio. It is possible that a wider range of P:C ratios and the use of less-digestible starch instead of sucrose contributed to the different results of the two studies.

Nutrients in the diet affect food intake and the activity, secretion, and gene expression of digestive enzymes. Enzymes can be induced by their substrate or suppressed by the reaction product. For example, the presence of lipids in an artificial diet induces the expression of lipase genes in the midgut of *Epiphyas postvittana* larvae [137] and secretion in *Gryllus bimaculatus* [128]. Glucose and maltose stimulate the secretion of amylase in the midgut of *Spodoptera frugiperda* [138]. The expression of amylase in *Drosophila melanogaster* is induced by starch and suppressed by dietary glucose [139,140], whereas amylase in *G. bimaculatus* is stimulated by glucose [127]. Interestingly, amylase in *Tribolium confusum* can be induced even in the absence of starch when the diet contains yeast as a protein source [141]. In addition to the induction of carbohydrase activities by starch, we also found a significant effect of dietary proteins on carbohydrases. On average, lower amylase and α-glucosidase activities were detected when dietary protein content was low, and this was even more evident when the starch content was also low. These responses may be due to the neuroendocrine regulation of enzyme activities and metabolic feedback mechanisms based on the metabolite level in the hemolymph [38], which allow the proteinase–amylase ratio to be adjusted to nutrient requirements. In addition, the activities of two carbohydrases showed a dependence of thermal reaction norms on the quality of the diet.

Coordinating the regulation of digestive enzymes and nutrient absorption is of paramount importance for a species that faces limited protein content in leaves and large variation in leaf nutrient ratios. Barbehenn et al. [95,123] found a strong relationship between the P:C ratio in the leaves of four Salicaceae plant species and the ratio of the protein-to-carbohydrate assimilation rate in SML. They suggested that SML employed post-absorptive mechanisms to achieve an appropriate ratio of assimilated nutrients. However, the present study confirmed that the homeostatic regulation of digestive enzyme activities also contributes to nutrient balancing. Coordinated enzyme activities were maintained across a range of experimental conditions, and only at 28 °C did a decrease in the nutrient content and P:C ratio alter the overall correlation structure of digestive enzymes. The SML growth rate was positively correlated with the utilization of proteins in oak leaves [93], while the ratio of the protein-to-carbohydrate assimilation rate in the range of 1–4 had no effect on the growth rate of SML fed Salicaceae leaves [123]. Our study found that reducing the P:C ratio below 1 significantly impaired larval growth and digestive physiology.

Similar to fitness-related traits, canonical discriminant analysis revealed the separation of temperature groups along the first canonical function and the separation of groups fed diets with different nutrient contents and ratios along the second canonical function. Thus, the differences in fitness traits between experimental treatments were associated with the differences in enzyme activities. Using multiple regression analysis, we found that the increased growth rate of SML was positively related to total protease and amylase activity, which was consistent with the results obtained in other insect species [129,142,143,144]. Depending on the insect species and the range of environments studied, the modulation of digestive enzyme activities may underlie constant growth in heterogeneous environments when a lack of correlations between fitness traits and enzyme activities is expected [24,31,134,145,146]. Interestingly, the SML growth rate and development rate (reciprocal of development duration) showed a strong negative relationship with lipase activity and a positive relationship with total protease. Under stressful conditions, increased energy demand can lead to a trade-off between the production of digestive enzymes that provide building blocks for growth and enzymes that provide energy resources. Similar responses have been observed in the presence of secondary metabolites in SML [147] and other pests (e.g., [148]).

## 5. Conclusions

In summary, the main findings of our study are as follows: (1) Temperature, the dietary nutrient content, and the dietary P:C ratio significantly affected the fitness-related traits and digestion of SM larvae; (2) The average thermal reaction norms were mostly similar among the different diets, although planned comparisons showed that the influence of diet quality was more prominent at certain temperatures; (3) Diet dilution decreased the activities of several enzymes, while the homeostatic regulation of proteases and carbohydrases was recorded in response to the altered P:C ratio; (4) The correlation structure of digestive enzymes changed in response to the reduced nutrient content and P:C ratio only at 28 °C; (5) Variations in the development duration, larval mass, and growth rate in response to temperature and dietary treatments were associated with variations in enzyme activities.

Our study confirmed that the midgut of SM can function as a nutrient-balancing organ by modulating digestive enzyme activities. The dependence of larval fitness-related traits on digestive enzyme activity points to the importance of a deeper understanding of the regulatory mechanisms of digestion. Here, we demonstrated temperature- and diet-induced plasticity of SM growth and digestion in the fourth instar. However, future studies should also consider advanced larval instars, as nutritional requirements and the ability to cope with multifactorial environmental variations may change during larval development and affect the final outcome at the level of SM pupal mass, adult reproductive capacity, population dynamics, and interactions with other species in the community. Investigating the interaction effects of temperature and diet on SM will help identify the potential consequences of projected global warming on the invasion potential of SM. As the first step, our laboratory study with artificial diets showed that larval development was accelerated at the highest temperature studied without detriment to body mass, but that, at the same time, the growth rate and coordination of enzyme activities were more affected by the low quality of the food. The significance of this result can be appreciated when considering that, in addition to temperature, leaf characteristics relevant to SM performance (e.g., toughness, nitrogen content, and defense compounds) may also be affected by climate change. Moreover, SM is exposed to daily temperature fluctuations under natural conditions. Therefore, to better mimic the real situation in the field, the next step should be to investigate the responses of SML to the combined effects of temperature fluctuations and a wider range of diets that differ not only in nutrient content and ratio but also in secondary metabolites.

## Figures and Tables

**Table 1:** Diet composition expressed as percentage of dry weight for high-protein–high-carbohydrate (HpHc), high-protein–low-carbohydrate (HpLc), low-protein–high-carbohydrate (LpHc), and low-protein–low-carbohydrate (LpLc) artificial diets.

Ingredient	HpHc	HpLc	LpHc	LpLc
Agar	8.34	8.34	8.34	8.34
Wheat germ	27.58	27.58	27.58	27.58
Casein	15.26	15.26	0	0
Potato starch	16.99	2.00	16.99	2.00
Alpha-cellulose	20.71	35.70	35.97	50.96
Wesson salt mix	4.45	4.45	4.45	4.45
Sorbic acid	1.11	1.11	1.11	1.11
Vanderzant vitamin mix	5.56	5.56	5.56	5.56

**Table 2:** Within canonical structure and standardized canonical coefficients for two discriminant functions obtained from discriminant analysis based on development duration and body mass. Loadings higher than 0.3 are given in bold.

	Within Groups Loadings		Standardized Coefficients	
Variable	Root 1	Root 2	Root 1	Root 2
Development duration	**−0.978**	−0.210	−0.953	−0.327
Larval mass	**+0.324**	**−0.946**	+0.211	−0.985

**Table 3:** Within canonical structure and standardized canonical coefficients for three discriminant functions obtained from discriminant analysis based on activities of digestive enzymes. Loadings above 0.3 are marked in bold.

	Within Groups Loadings			Standardized Coefficients		
Variable	Root 1	Root 2	Root 3	Root 1	Root 2	Root 3
PA	+0.080	−0.132	**+0.391**	+0.496	−1.055	−0.276
TRY	+0.015	−0.040	**+0.567**	−0.200	−0.469	+1.391
ELA	+0.131	+0.181	+0.155	+0.382	+0.988	−0.770
LAP	**−0.777**	−0.002	+0.056	−1.156	−0.116	−0.286
AMY	−0.082	**+0.517**	**+0.318**	+0.171	+0.905	+0.158
α-GLUC	−0.184	+0.136	**+0.694**	−0.197	+0.372	+0.581
LIP	−0.027	−0.284	+0.028	+0.264	−0.510	+0.011

PA—protease activity, TRY—trypsin, ELA—elastase, LAP—leucine aminopeptidase, AMY—α-amylase, α-GLUC—α-glucosidase. LIP—lipase.

**Table 4:** Multiple stepwise regression analysis relating development time and body mass of spongy moth larvae to digestive enzyme activity variation in response to temperature and diet quality.

Response Variable	Predictor ^1^	β ± SE	t	*p*	Adjusted R^2^, Whole Model
Development	PA	−0.759 ± 0.150	−5.06	0.002	0.950
duration	TRY	+0.286 ± 0.114	2.51	0.046	*p* < 0.001
	LAP	+0.345 ± 0.121	2.85	0.029	
	α-GLUC	+0.283 ± 0.114	2.48	0.048	
	LIP	+0.894 ± 0.122	7.31	<0.001	
Body mass	LAP	−0.443 ± 0.049	−9.07	<0.001	0.977
	AMY	+0.414 ± 0.071	5.82	<0.001	*p* < 0.001
	LIP	−0.505 ± 0.072	7.01	<0.001	
Growth rate	PA	+0.684 ± 0.146	4.69	0.003	0.935
	TRY	−0.312 ± 0.130	−2.39	0.054	*p* < 0.001
	AMY	+0.317 ± 0.133	2.39	0.054	
	α-GLUC	−0.501 ± 0.103	−4.85	0.003	
	LIP	−0.973 ± 0.135	−7.23	<0.001	

^1^ PA—protease activity, TRY—trypsin, LAP—leucine aminopeptidase, AMY—α-amylase, α-GLUC—α-glucosidase, LIP—lipase.

## Data Availability

The data presented in this study are available upon request from the corresponding author.

## Supplementary Materials

The following supporting information can be downloaded at: https://www.mdpi.com/article/10.3390/biom13050821/s1, Table S1: Results of three-way multivariate and univariate analyses of variance (F and *p* values) assessing main and interaction effects of temperature (T), dietary protein (P), and carbohydrate content (C) on larval fitness-related traits. Significant effects are marked in bold; Table S2: Significance of squared Mahalanobis distances (D^2^) between treatment groups for fitness-related traits; Table S3: Results of three-way multivariate and univariate analyses of variance assessing main and interaction effects of temperature (T), dietary protein (P), and carbohydrate content (C) on digestive enzyme activities. Significant effects are marked in bold; Table S4: Results of z test for comparisons of specific correlation coefficients and Mantel test for comparison of correlation structures between digestive enzyme activities in larvae reared at (A) different temperatures (19 °C vs. 23 °C, 23 °C vs. 28 °C) within each diet and (B) different diets (HpHc vs. LpLc, HpLc vs. LpHc) within each temperature. Significant differences between specific correlations are presented in red; Table S5: Significance of squared Mahalanobis distances (D^2^) between treatment groups for digestive enzyme activities.
