# Supplementary material for: Temperature- and Diet-Induced Plasticity of Growth and Digestive Enzymes Activity in Spongy Moth Larvae
Source: Biomolecules. 2023 May 11;13(5):821. doi: 10.3390/biom13050821 (PMC10216847; doi:10.3390/biom13050821)
Supplement: Supplementary file 1 [file biomolecules-13-00821-s001.zip › biomolecules-2348712-supplementary.pdf]

Table S1. Results of three-way multivariate and univariate analyses of variance (F and *p* values) assessing main and interaction effects of temperature (T), dietary protein (P) and carbohydrate content (C) on larval fitness-related traits. Significant effects are marked in bold.

	**MANOVA**				**Dev. duration**		**Larval mass**	
	**df, df_{error}**	**F**	***p***	**df, df_{error}**	**F**	***p***	**F**	***p***
T	4, 336	68.21	**< 0.001**	2, 168	463.61	**< 0.001**	43.45	**< 0.001**
P	2, 167	34.14	**< 0.001**	1, 168	10.47	**0.001**	63.37	**< 0.001**
C	2, 167	2.54	*0.082*	1, 168	0.01	0.911	4.98	**0.027**
T × P	4, 336	0.06	0.994	2, 168	0.01	0.995	0.10	0.902
T × C	4, 336	0.58	0.678	2, 168	0.12	0.890	0.96	0.387
P × C	2, 167	2.94	*0.056*	1, 168	1.74	0.190	4.77	**0.030**
T × P × C	4, 336	0.41	0.802	2, 168	0.61	0.543	0.22	0.801

Table S2. Significance of squared Mahalanobis distances (D^2) between treatment groups for fitness-related traits.

A.

Diet		19 °C vs. 23 °C	23 °C vs. 28 °C
HpHc	D^2	15.94	6.55
	F	58.35	23.97
	p	**< 0.001**	**< 0.001**
HpLc	D^2	13.33	9.25
	F	48.81	33.87
	p	**< 0.001**	**< 0.001**
LpHc	D^2	9.89	6.82
	F	36.20	24.98
	p	**< 0.001**	**< 0.001**
LpLc	D^2	7.97	4.05
	F	29.19	14.82
	p	**< 0.001**	**< 0.001**

B.

Temperature		HpHc vs. LpLc	HpLc vs. LpHc
19 °C	D^2	0.84	2.33
	F	3.11	8.57
	p	0.053	**< 0.001**
23 °C	D^2	0.60	3.94
	F	2.20	14.49
	p	0.120	**< 0.001**
28 °C	D^2	1.29	1.83
	F	4.73	6.76
	p	**0.013**	**0.002**

Table S3. Results of three-way multivariate and univariate analyses of variance assessing main and interaction effects of temperature (T), dietary protein (P) and carbohydrate content (C) on digestive enzyme activities. Significant effects are marked in bold.

	**MANOVA**				**PA**		**TRY**		**ELA**		**LAP**		**AMY**		**α-GLUC**		**LIP**	
	**df, df_{error}**	**F**	***p***	**df, df_{error}**	**F**	***p***	**F**	***p***	**F**	***p***	**F**	***p***	**F**	***p***	**F**	***p***	**F**	***p***
T	14, 326	16.44	**<0.001**	2, 168	0.87	0.420	3.33	**0.038**	6.71	**0.002**	107.08	**<0.001**	0.66	0.518	8.27	**<0.001**	2.60	*0.077*
P	7, 162	39.91	**<0.001**	1, 168	6.12	**0.014**	0.31	0.577	5.59	**0.019**	24.81	**<0.001**	75.61	**<0.001**	8.81	**0.003**	19.19	**<0.001**
C	7, 162	2.53	**0.017**	1, 168	1.85	0.175	3.45	*0.065*	0.60	0.439	8.55	**0.004**	0.37	0.544	0.98	0.323	0.12	0.725
T×P	14, 326	1.76	**0.043**	2, 168	2.68	*0.071*	0.77	0.465	1.39	0.253	0.43	0.654	2.13	0.122	0.85	0.427	1.2	0.303
T×C	14, 326	1.66	*0.063*	2, 168	2.34	*0.099*	0.94	0.395	0.44	0.645	2.26	0.108	4.34	**0.014**	3.45	**0.034**	0.00	0.998
P×C	7, 162	1.38	0.217	1, 168	0.44	0.510	0.80	0.371	0.25	0.616	1.42	0.235	1.23	0.269	1.22	0.270	0.29	0.594
T×P×C	14, 326	0.59	0.870	2, 168	0.20	0.822	0.01	0.987	0.22	0.801	0.07	0.928	0.30	0.738	1.06	0.349	0.27	0.765

Table S4. Results of z test for comparisons of specific correlation coefficients and Mantel test for comparison of correlation structures between digestive enzyme activities in larvae reared at (A.) different temperatrures (19 °C vs. 23 °C, 23 °C vs. 28 °C) within each diet and (B.) different diets (HpHc vs. LpLc, HpLc vs. LpHc) within each temperature. Significant differences between specific correlations are presented in red.

A.

	**HpHc**		**HpLc**		**LpHc**		**LpLc**	
	**19 °C vs. 23 °C**	**23 °C vs. 28 °C**	**19 °C vs. 23 °C**	**23 °C vs. 28 °C**	**19 °C vs. 23 °C**	**23 °C vs. 28 °C**	**19 °C vs. 23 °C**	**23 °C vs. 28 °C**
PA-TRY	-0.21	-0.04	1.30	0.47	-0.06	0.83	-0.27	0.68
PA-ELA	-1.62	0.47	2.05	0.36	-0.32	0.28	-0.27	2.38
PA-LAP	-1.43	-0.84	0.76	-0.05	-0.09	1.46	0.86	1.90
PA-AMY	-0.18	-0.72	-0.04	-0.66	-0.22	0.79	1.15	-0.19
PA-GLUC	-1.28	-0.19	0.26	0.90	0.45	-0.37	-2.78	2.30
PA-LIP	0.55	-1.47	0.21	-0.57	-0.02	1.61	0.54	0.48
TRY-ELA	-1.59	-0.54	-0.58	0.91	-0.44	-0.22	-0.61	2.19
TRY-LAP	-1.27	-1.45	0.09	-0.91	-0.39	1.41	1.36	1.78
TRY-AMY	-1.09	-0.11	-0.10	-1.11	-0.18	0.62	1.80	-0.30
TRY-GLUC	-1.04	0.02	1.03	-0.49	0.36	-0.75	-2.26	1.14
TRY-LIP	0.00	-0.48	-0.63	-0.32	-0.37	1.40	0.49	-0.28
ELA-LAP	-0.69	-2.07	0.98	-1.82	-0.22	0.80	0.65	1.19
ELA-AMY	-1.38	-0.11	0.46	-2.48	-0.10	0.23	1.20	-1.10
ELA-GLUC	-1.29	-0.21	-0.72	-0.52	0.93	-0.32	-2.14	2.35
ELA-LIP	-0.03	-0.21	-1.12	-0.59	-0.54	1.34	1.10	-0.79
LAP-AMY	-0.32	-1.92	-1.85	0.24	0.89	0.15	1.89	-1.20
LAP-GLUC	-1.77	1.38	0.19	-0.95	1.15	-0.61	-0.67	1.51
LAP-LIP	1.57	-1.13	-0.90	-0.05	0.92	0.07	0.53	-1.20
AMY-GLUC	-0.70	-0.62	0.42	-0.34	-0.04	-1.07	1.14	0.43
AMY-LIP	0.19	-1.35	0.47	0.03	0.17	-0.33	-0.24	-0.64
GLUC-LIP	3.28	-1.25	-1.18	0.16	-0.55	1.66	1.15	0.06
Mantel:								
R	0.481	0.744	0.373	0.327	0.708	0.485	0.184	0.079
p	**<0.001**	**<0.001**	**0.002**	**0.004**	**<0.001**	**<0.001**	**0.024**	0.146

PA - protease activity, TRY – trypsin, ELA – elastase, LAP – leucine aminopeptidase, AMY – α-amylase, α-GLUC - α-glucosidase, LIP - lipase

continue…

B.

	19 °C		23 °C		28 °C	
	**HpHc vs. LpLc**	**HpLc vs. LpHc**	**HpHc vs. LpLc**	**HpLc vs. LpHc**	**HpHc vs. LpLc**	**HpLc vs. LpHc**
PA-TRY	-0.14	-1.53	-0.20	-2.88	0.52	-2.52
PA-ELA	-1.03	-0.09	0.32	-2.46	2.22	-2.54
PA-LAP	-1.27	0.46	1.02	-0.39	**3.77**	1.12
PA-AMY	0.70	0.24	2.04	0.06	2.57	1.51
PA-GLUC	-0.23	0.41	-1.73	0.61	0.76	-0.67
PA-LIP	-0.99	-0.34	-0.99	-0.58	0.96	1.60
TRY-ELA	-0.93	-0.82	0.05	-0.67	2.78	-1.81
TRY-LAP	-2.41	0.16	0.23	-0.31	**3.46**	2.00
TRY-AMY	-0.05	0.16	2.84	0.08	2.64	1.81
TRY-GLUC	-0.13	1.45	-1.35	0.78	-0.23	0.52
TRY-LIP	-0.86	0.04	-0.36	0.30	-0.16	2.02
ELA-LAP	-1.77	0.86	-0.44	-0.34	2.82	2.28
ELA-AMY	0.72	-0.05	**3.30**	-0.62	2.31	2.08
ELA-GLUC	-1.01	-0.61	-1.86	1.04	0.70	1.23
ELA-LIP	-1.58	-0.31	-0.45	0.28	-1.03	2.21
LAP-AMY	-1.40	-1.71	0.80	1.02	1.53	0.93
LAP-GLUC	-0.49	-0.07	0.61	0.89	0.74	1.24
LAP-LIP	-0.64	-0.90	-1.68	0.91	-1.75	1.03
AMY-GLUC	-1.83	0.70	0.01	0.24	1.06	-0.48
AMY-LIP	-1.15	0.99	-1.58	0.70	-0.86	0.34
GLUC-LIP	1.35	-0.79	-0.79	-0.15	0.52	1.35
Mantel:						
R	0.484	0.563	0.263	0.563	0.044	0.130
p	**<0.001**	**<0.001**	**0.006**	**<0.001**	0.252	0.115

PA - protease activity, TRY – trypsin, ELA – elastase, LAP – leucine aminopeptidase, AMY – α-amylase, α-GLUC - α-glucosidase, LIP - lipase

Table S5. Significance of squared Mahalanobis distances (D^2) between treatment groups for digestive enzyme activities.

A.

Diet		19 °C vs. 23 °C	23 °C vs. 28 °C
HpHc	D^2	11.14	1.66
	F	10.23	1.52
	p	**< 0.001**	0.192
HpLc	D^2	11.99	1.69
	F	11.01	1.55
	p	**< 0.001**	0.182
LpHc	D^2	6.60	6.11
	F	6.06	5.62
	p	**< 0.001**	**< 0.001**
LpLc	D^2	7.10	4.76
	F	6.52	4.37
	p	**< 0.001**	**0.001**

B.

Temperature		HpHc vs. LpLc	HpLc vs. LpHc
19 °C	D^2	12.37	5.87
	F	11.84	5.62
	p	**< 0.001**	**< 0.001**
23 °C	D^2	7.26	17.60
	F	6.94	16.84
	p	**< 0.001**	**< 0.001**
28 °C	D^2	7.27	6.83
	F	6.96	6.53
	p	**< 0.001**	**< 0.001**
